# Crossmodal correspondences between visual and speech angularity and tactile jaggedness of response key

**DOI:** 10.1038/s41598-024-79400-4

**Published:** 2024-11-13

**Authors:** Yusuke Suzuki, Naoki Ueno, Keigo Nakahashi, Masayoshi Nagai

**Affiliations:** 1 Graduate School of Human Science, Ritsumeikan University, Osaka Ibaraki Campus Ritsumeikan University, 2-150, Iwakuracho, Ibaraki, Osaka 567-8570 Japan; 2 Japan Society for the Promotion of Science (JSPS), Tokyo, 102-0083 Japan; 3 Department of Comprehensive Psychology, Ritsumeikan University, Ibaraki, 567-8570 Japan

**Keywords:** Crossmodal correspondences, Sound symbolism, Stimulus-response compatibility, Psychology, Human behaviour

## Abstract

Several studies reported various crossmodal correspondences related to tactile features. These previous studies have investigated tactile-related correspondences through explicit matching or subjective evaluation tasks, which required participants to recognize relationships between tactile and other sensory features or rate tactile materials on scales with adjective labels related to visual or auditory features. However, these tasks are prone to occur the experimenter-expectancy effects and arbitrary categorization of tactile materials by the labels, making it difficult to assess implicit and non-arbitrary aspects of crossmodal correspondences. To address this, we used a speeded classification task to examine whether the angularity/roundedness of visual and auditory stimuli correspond to tactile jaggedness/fluffiness. Participants distinguished between angularity or roundedness (Experiment 1: visual shapes; Experiment 2: speech sounds) by pressing right- or left-positioned response keys with task-irrelevant jaggedness or fluffiness without prior instruction on which key represented jaggedness/fluffiness. Results showed faster keypresses for jagged/fluffy responses to angular/rounded stimuli, suggesting an implicit correspondence between these sensory features except for the experimenter-expectancy effects and the influence of the labels. Unlike previous studies that examined the correspondence with simple tactile features (e.g., weight, size), our findings suggest that even complex tactile-quality features, such as jaggedness/fluffiness, implicitly correspond to visual and auditory angularity/roundedness.

**Supplementary Information:**

The online version contains supplementary material available at 10.1038/s41598-024-79400-4.

## Introduction

People can predict an object’s shape, size, and fluffiness without even touching it if they can see or listen to it. When we see a wool muffler, we anticipate that it will be soft and warm. When we hear sounds of high heels while walking, we can predict that the floor is hard and cold. Our brain exhibits a tendency toward non-arbitrary associated features and dimensions of these unisensory signals, a phenomenon known as *crossmodal correspondence*^1^. *Sound symbolism*, the non-arbitrary correspondence between syllable sequences and particular meanings, is a typical example. Köhler^2^ suggested a famous example of sound symbolism and demonstrated that participants tended to associate nonsense words, *takete*/*maluma*, and visual drawings of angular/rounded shapes. Nonsense words, which included fricative consonants (*takete*), were associated with angular shapes. While many studies focused on the correspondence between visual and auditory dimensions, some addressed correspondences related to other modalities’ dimensions (audio-taste:^3^; smell-audio and visual:^4,5^).

Several studies reported correspondences in the tactile dimension. Tactile dimensions (weight, smoothness, and hardness) were reportedly associated with some visual (luminance and angularity) and auditory dimensions (pitch, intensity)^5–11^. Walker and Smith^12^ revealed that auditory tones with high/low pitch were identified as small/big and light/heavy and subsequently facilitated the identification of corresponding words (“big” and “heavy”) when low-pitch tones were presented. Walker et al.^13^ reported that when participants estimated the relative weights of objects without touching them, darker objects were heavier than brighter ones. These findings suggested that information about object properties obtained through tactile perception (weight, size) corresponded with another sensory feature obtained through other modalities. Humans can perceive and differentiate object properties, such as weight or size, and various *tactile-quality* features of an object, such as jaggedness, hardness, and roughness, from experiences. These tactile-quality features were not a single property of an object, such as weight and size; however, these were derived from the integration of multiple multisensory object properties. Several studies also supported crossmodal correspondence that involved tactile-quality features. Etzi et al.^14^ demonstrated that smoother tactile textures were associated with high-luminance, high-chromatic saturation color, low-pitch, low-intensity sounds, and rounded-sounding words, such as *maluma* in the tactile evaluation of real materials (satin and sandpaper). Studies on sound symbolism reported correspondences related to both tactile features. Lo et al.^15^ reported that sound symbolism was related to tactile-quality features via evaluation tasks. They demonstrated that fricative consonant speech corresponded to rough tactile surfaces. These studies suggested that correspondence occurred between visual, auditory, and tactile-quality features. Considering previous results on object properties, both types of object features were associated with the features of other modalities.

Research on crossmodal correspondence related to tactile features has employed explicit matching tasks between tactile objects and other sensory features or evaluation tasks for tactile objects. These tasks require explicit judgment regarding crossmodal features of the object. For example, in an explicit matching task, participants were presented with visual or auditory stimuli that had opposing features (e.g., an angular visual shape on the left side and a rounded visual shape on the right side of a screen). After viewing or listening to the stimuli, they were asked to touch a tactile material and choose either of the visual or auditory stimuli that was associated with the tactile material. In an evaluation task, participants touched various tactile materials and rated them on scales with adjective labels related to visual or auditory features (e.g., “dark” on the left and “light” on the right side of the scale). In these tasks, participants could categorize the tactile material and could notice the relationship between the tactile material’s feature and another sensory stimulus’s feature. Several studies^16,17^ discussed that such experimental procedures had several interpretational limitations and rarely implied the implicit or non-arbitrary correspondence. An experimenter-expectancy effect and the influence of linguistic/verbal labels are the most critical problems. Explicit judgment procedures make it easy to inform participants regarding what the experimenter intends to investigate. Thus, participants may have responded based on how they expected the experimenter wished them to respond^18^. Moreover, in the evaluation and matching tasks, individual sensory feature values are given linguistic labels on the rating scales. Labels influence rating behaviors^19^ and participants could confuse the association between the sensory features indicated by the label and tactile features of an evaluated object with the association between the label name and evaluated object and additionally might categorize the material’s features based on the labels, ignoring other features not expressed in labels. Several studies mentioned that the experimenter-expectancy effect^16,20,21^ and linguistic labels^22–25^ could influence object processing and crossmodal processing. In contrast, a speeded classification task could minimize the influence of the experimenter-expectancy effect and linguistic labels. In this task, participants are required to quickly and accurately distinguish a sensory feature in the presence of another task-irrelevant sensory feature. In such tasks, the experimenter does not need to instruct participants regarding the task-irrelevant sensory features. Additionally, the task-irrelevant features are not verbally labeled. Therefore, participants could neither guess the relationship between the stimulus’s feature and the task-irrelevant sensory features nor categorize the task-irrelevant features, thus excluding the experimenter-expectancy effect and the influence of labels in such task. Regarding this task, discrepancies in reaction times and error rates between the combinations of sensory features confirm the presence of a crossmodal correspondence^26^. Quick and accurate responses indicate a crossmodal correspondence between distinguished sensory features and task-irrelevant sensory features.

Some of these crossmodal correspondences have been found with speeded classification tasks as well as explicit judgment tasks. Walker and Walker^27^ demonstrated that the weight of response keys perceived through touch corresponded to the brightness of the visual stimulus via a speeded classification task. Participants classified brighter/darker objects by pressing the left and right keys, which were smaller/bigger keys. Under the compatible conditions, smaller/bigger keys were assigned to classify brighter/darker stimulus, whereas under the incompatible conditions, bigger/smaller keys were assigned. They demonstrated faster reaction times under the compatible than incompatible conditions. Participants classified the stimulus based on the left or right positions of the response keys, and difference in sizes was not instructed. Thus, their experiment procedure minimized any influence of the experimenter-expectancy effect and linguistic labels. Walker et al.^17^ introduced light/heavy response keys via a procedure similar to that of Walker and Walker^27^ and demonstrated a weight–brightness correspondence. These results suggested that the brightness stimulus primed participants to press the key with the corresponding object properties of the response key (size or weight). These findings suggested crossmodal correspondences occurred in a more implicit mode than those shown in previous studies that used explicit judgment tasks. Additionally, their findings implied that classified stimulus features (stimulus brightness and positions of the keys) and task-irrelevant properties of response keys (size or weight of the keys) were incorporated into a stimulus-response processing and involved in correspondences. However, it was unclear whether correspondences related to tactile-quality features could also be confirmed via a speeded classification task without explicit judgment of that. Hence, whether the tactile-quality features (jaggedness, hardness) of response keys correspond to distinguished stimulus features remains unclear.

We focused on crossmodal correspondences related to tactile-quality features, that is, the correspondence between visual and speech angularity and tactile hardness or jaggedness. This study investigated whether crossmodal correspondences related to tactile-quality features could occur even when a speeded classification task was used without the need for explicit matching of correspondent sensory features. We created a different response key and manipulate tactile-quality features similar to those accompanying motor responses. We examined the correspondence between these features using response keys covered with real materials of different tactile qualities. Similar to in previous studies, use of a response key to manipulate a simple single tactile feature, such as size or weight, was appropriate. Since the tactile-quality feature comprised multiple tactile features^14,28^, a response key with real materials that expressed multiple tactile-quality features was used in this study. We used the “jagged” and “fluffy” keys, which were expressed as jagged or hard and fluffy or soft, respectively. According to Lynott and Connell^29^, to evaluate each adjective and how it was experienced through each perceptual modality, we adopted tactile features (hard, jagged, soft, fluffy) to better express the response keys. Recent studies revealed that angular/rounded visual shapes and speech sounds were associated with tactile-quality features, such as hardness and roughness, through questionnaires and ratings of real materials, which included explicit judgment/identification of sensory features (visual-tactile:^11,14,30^; speech/auditory-tactile:^6,8,14–16^). Therefore, this study examined these correspondences via a speeded classification task. In Experiments 1 and 2, we examined the correspondence between the visual shape and tactile-quality features and between speech sounds, such as symbolic words, and tactile-quality features, respectively. In both experiments, the reaction times (RT) and error rates were measured and calculated the inverse efficiency score (IES)^31^ to combine speed and error. In tasks that required both speed and accuracy of responses, such as the speeded classification task, experimental effects could appear in some participants in only in RT (speed) or error rate (accuracy) owing to the speed-accuracy trade-off. When ignoring one behavioral measure and drawing conclusions based solely on another measure can lead to erroneous interpretations although the observed effects merely reflect the speed-accuracy trade-off. An often-suggested solution to the trade-off was the IES, which integrated speed and accuracy into a single measure^32–34^. The IES was calculated by dividing the averaged RT by the proportion of correct responses (1 – error rates), thereby weighting speed according to accuracy. When two conditions exhibited the same average RT although differing error rates, the IES of the condition with fewer error trials would further decrease compared with the IES of the condition with more error trials. Therefore, the lower IES indicated that participant’s response was quicker and more accurate than the higher IES. Regarding this study, if the correspondences occurred in the speeded classification tasks as with previous studies that used tasks with explicit judgment, the IES was predicted to be lower in a combination of angular/rounded stimulus and jagged/fluffy keypress than in an opposite combination.

## Experiment 1

We examined the crossmodal correspondence between visual and tactile-quality features. Participants judged the shape of a visual stimulus by pressing left or right alternative response keys with different tactile-quality features. We did not mention the tactile-quality features to participants to minimize any influence of experimenter-expectancy effect and linguistic labels of the tactile-quality feature.

## Methods

### Participants

We recruited 16 undergraduate and graduate students from Ritsumeikan University who reported normal or corrected-to-normal vision for Experiment 1 (*M*_age_ = 21.00 years, range 19–23 years, six females). All the participants provided written informed consent. The sample size was determined based on the results of the power analysis and counterbalanced. A pilot study that preceded Experiment 1 obtained interaction effect sizes that corresponded roughly to large effect size (*f* = 0.514, details in the supplementary materials). Therefore, a power analysis was used and G*Power3.1.9.6^35^ assumed a large effect size (*f* = 0.40, based on Cohen^36^), significance level of 5% (α = 0.05), and power of 0.95 (1–β = 0.95), which aimed to detect the interaction in a repeated-measures analysis of variance (ANOVA) within the participants. The experiment was conducted according to the guidelines of the Declaration of Helsinki. The experimental procedure was approved by the Research Ethics Review Committee of the Department of Comprehensive Psychology and Graduate School of Human Science at Ritsumeikan University.

### Apparatus and stimuli

The experiment was conducted via PsychoPy Buider3^37^ that was run on a PC (Dell, XPS15 9560). Visual stimuli were presented at the center of the LCD (BenQ, XL2411P, 24 inch and a resolution of 1920 × 1080 pixels). The observation distance was approximately 60 cm and was fixed via a chin stand. The visual stimuli and background were white and gray, respectively.

Studies on sound symbolism have used angular and rounded shapes^2,38^. These shapes were made according to Walker^11^, and five for each shape (total of 10 shapes in Supplement Fig. 2a) were selected for the present experiment based on subjective evaluation scores with independent raters (7-point scales that referred to “rounded - angular”: 1 for extremely rounded; 7 for extremely angular). The dimensions of each visual shape were 8.4° × 8.4°. Two flat response keys (TechnoWave, OFL-SG5-H-MCA-1-1.6 M, diameter 9 cm) were used to obtain participants’ manual responses. One key covered with jagged pumice stones was defined as the “jagged” key, and the other covered with a piece of blanket was defined as the “fluffy” key. Both keys were placed in a cardboard box and a black cloth prevented participants from seeing the keys and assuming their tactile features through visual information (Supplementary Fig. 2b, c). Response keys were placed on the left and right sides. After the experiment, participants evaluated the response keys on five dimensions (tactile impression: “soft,” “hard,” “fluffy,” “jagged,” “ease of key press”). Since real objects cannot be expressed only as a single feature dimension (e.g^14,28^), items on tactile impression comprised four adjectives (tactile impression: 1 = not at all, 9 = extremely applicable; ease of key press: 1 = extremely hard, 9 = extremely easy). Tactile impression words were obtained from Lynott and Connell^29^. Table 1 lists the average values calculated for each dimension. We conducted a paired two-sided *t-*test (Bonferroni-corrected, *p* < .05 prior to correction). Consequently, a significant difference was observed between the response keys in all the dimensions (jaggedness: *t*(15) = 92.223, *p* < .001, *d* = 23.056; fluffiness: *t*(15) = 57.455, *p* < .001, *d* = 14.364; softness: *t*(15) = 57.455, *p* < .001, *d* = 14.364; hardness: *t*(15) = 40.021, *p* < .001, *d* = 10.005, all comparisons were significant at *p* < .01). However, no significance was observed for ease of pressing key (*t*(15) = 2.452, *p* = .027, *d* = 0.613). This result showed that one key had fluffy and soft features, whereas the other key had jagged and hard features. Therefore, each word had the highest strength rating for the tactile modality and was adequate for expressing the response keys.

**Table 1:** Answers to questionnaires about response keys.

	Jaggedness	Fluffiness	Hardness	Softness	Ease of pressing key
Jagged key	8.875 (0.085)	1.000 (0.000)	8.938 (0.063)	1.000 (0.000)	4.500 (0.639)
Fluffy key	1.000 (0.000)	8.813 (0.136)	1.188 (0.136)	8.813 (0.136)	6.813 (0.476)

Standard errors are indicated within parentheses. Items with a standard error of 0 suggest that all participants provided the same evaluation.

### Procedures

The experimental design had two within-subject factors: visual shapes (angular or rounded) and tactile features of the response keys (jagged or fluffy). Participants were required to classify angular or rounded shapes by pressing either the left or right key as quickly and accurately as possible. In some blocks, participants were instructed to press the right key for angular visual stimuli and the left key for rounded visual stimuli, while in other blocks, this combination was reversed (i.e., the right key for the rounded stimuli and the left key for the angular stimuli). The keys had either jagged or fluffy tactile-quality surfaces, respectively, and were not visually presented (black cloth was put over them). These tactile features of the keys were task-irrelevant, and participants were not instructed to press the jagged or fluffy key and only press the left or right key. Therefore, participants had no information on which key was jagged or fluffy. In addition, participants were instructed not to touch both response keys before the visual stimulus was presented to avoid adaptation to the tactile-quality feature.

Participants completed four blocks of 40 trials each. Each block was assigned an experimental condition: either a compatible (jagged/fluffy key for an angular/rounded visual stimulus) or an incompatible condition (jagged/fluffy key for a rounded or angular visual stimulus). In each block and practice trials, 40 (10 shapes × 4 different angles: 0°, 90°, 180°, and 270°) and 10 visual stimuli (10 shapes at a fixed 0° angle) were presented in random order, respectively. The position (left or right) of each key was changed between the blocks, if necessary. In each trial, a blank screen was presented for 500 ms, and the visual stimulus was subsequently presented until participants responded. The order of the experimental conditions and positions of the response keys were counterbalanced.

## Results

RT was defined as the duration between the onset of the visual stimulus presentation and timing of pressing the key. Trials where RT was less than more than three standard deviations from each participant’s average RT (2.46% of trials) were labelled as outliers and excluded. Error trial was defined as a trial in which a response was made on a different side of key with requirements of the experimental condition in each trial. Additionally, trials in which response errors were made (2.31% of trials) were excluded. We calculated the IES to combine speed and accuracy. The IES was calculated by dividing the averaged RT by the proportion of correct responses (PC: 1-error rates).

**Figure 1:**
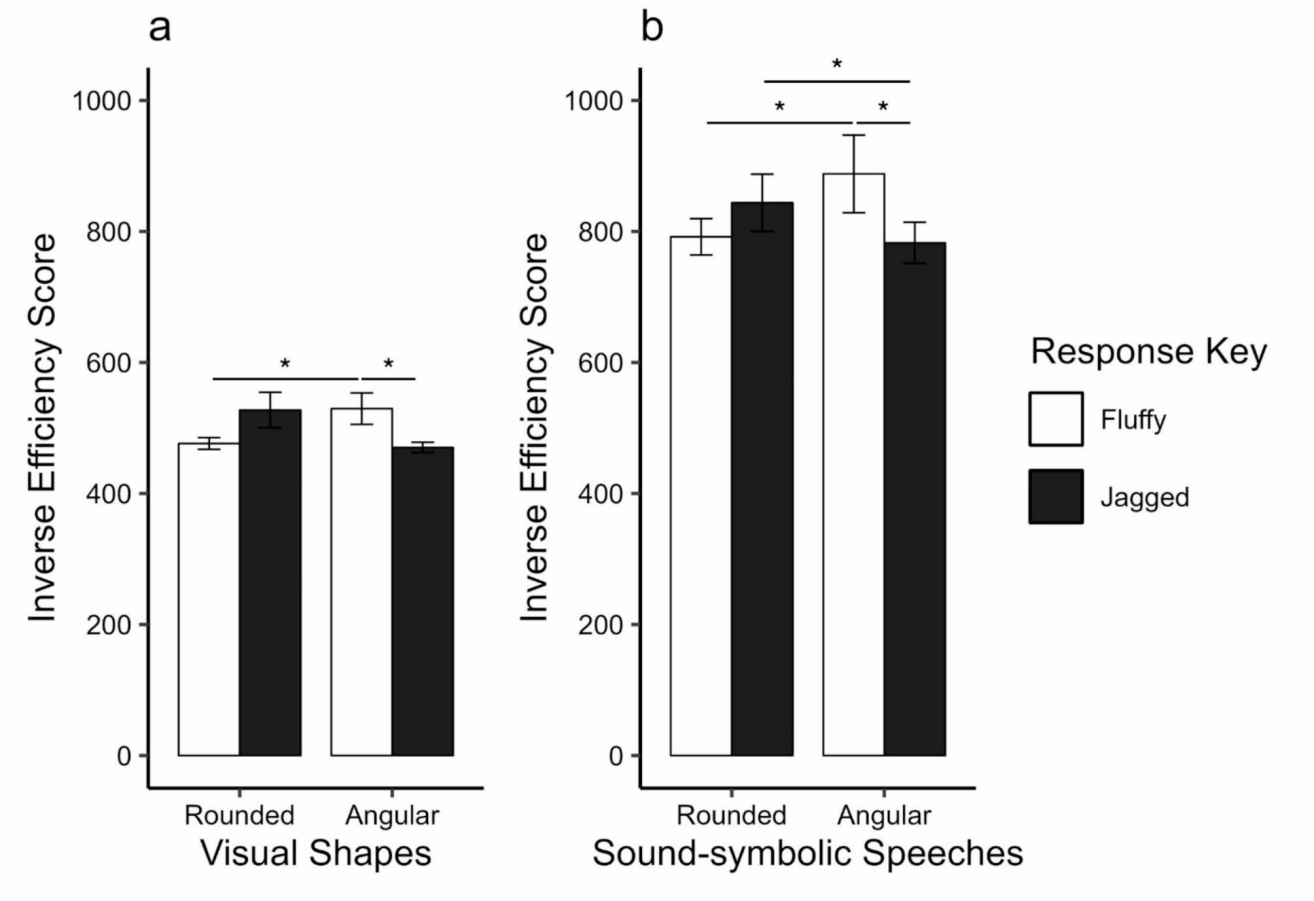
(**a**) Mean IESs for visual shapes × tactile-quality features of response key; (**b**) Mean IESs for sound-symbolic speeches × tactile-quality features of response key. Error bars indicate standard errors.

Average IES was calculated for each participant for each combination of the visual and tactile features (Fig. 1a). Responses of the jagged keys to angular visual stimuli were faster and more accurate (*M*_RT_ = 460.748 ms, *M*_PC_ = 97.969%) than to rounded visual stimuli (*M*_RT_ = 514.002 ms, *M*_PC_ = 97.500%). In contrast, the responses of the fluffy keys to rounded visual stimuli were faster and more accurate (*M*_RT_ = 463.938 ms, *M*_PC_ = 97.500%) than to angular visual stimuli (*M*_RT_ = 513.848 ms, *M*_PC_ = 97.188%). We conducted a 2 (visual shape: angular versus rounded) × 2 (tactile feature: jagged versus fluffy) ANOVA. Consequently, a significant interaction was observed between visual shapes and tactile features (*F*(1,15) = 4.790, *p* = .045, η_p_^2^ = 0.242). Both main effects of visual shapes (*F*(1,15) = 0.157, *p* = .697, η_p_^2^ = 0.010) and tactile features (*F*(1,15) = 0.722, *p* = .409, η_p_^2^ = 0.046) were not significant. Since the interaction was significant, we conducted simple effects tests. Keypresses to the jagged key for the angular shapes were faster than those to the fluffy key for the angular shapes (*F*(1,15) = 6.348, *p* = .024, η_p_^2^ = 0.297). Keypresses to the fluffy key for the rounded shapes were faster than those to the fluffy key for the angular shapes (*F*(1,15) = 4.813, *p* = .044, η_p_^2^ = 0.243). However, there were no differences in the IES between the fluffy and jagged keys for the rounded shapes (*F*(1,15) = 3.432, *p* = .084, η_p_^2^ = 0.186) and angular and rounded shapes for the jagged key (*F*(1,15) = 4.467, *p* = .052, η_p_^2^ = 0.230).

Results of Experiment 1 revealed that the IES for pressing the fluffy key on rounded shapes was lower than for angular shapes. This result suggested a correspondence between visual shape and tactile-quality features, in particular, fluffy/soft tactile-quality features and rounded visual shapes. Hence, this experiment revealed the correspondence without explicit judgement of tactile-quality feature via speeded classification task. However, there was no difference in the IES with the jagged key. Perhaps the 3D shapes of pumice stones of the jagged key influenced these results. These stones typically have round 3D shapes at the macroscopic level. Therefore, participants could have associated the rounded shapes of the pumice stones with the rounded visual shapes. Thus, such an association might conflict with the correspondence between angular visual shape and jagged tactile-quality feature.

### Experiment 2

Lo et al.^15^ reported a sound symbolism between sound-symbolic speeches (e.g., “*Bouba*,” “*Kiki*”) and smooth/rough tactile-quality feature. They conducted rating experiments and identified fricative consonant syllables associated with rough-surface materials. In Experiment 2, we examined the crossmodal correspondence between rounded/angular sound-symbolic words and fluffy/jagged keys via a speeded classification task without explicit judgment of the tactile-quality features.

## Methods

### Participants

An additional 16 undergraduate and graduate students from Ritsumeikan University (*M*_age_ = 21.63 years, range = 19–23 years, six females) who reported normal or corrected-to-normal vision were recruited for Experiment 2. All the participants provided written informed consent. The sample size was set equal to that of Experiment 1 as the experimental design was the same. The experiment was conducted according to the guidelines of the Declaration of Helsinki. The experimental procedure was approved by the Research Ethics Review Committee of the Department of Comprehensive Psychology and Graduate School of Human Science at Ritsumeikan University.

### Apparatus and stimuli

The apparatus used in Experiment 2 was the same as that used in Experiment 1, except for the presentation of speech sound stimuli. In Experiment 2, we used a speech stimulus instead of a visual stimulus. Therefore, we recorded eight nonsense words vocalized by two volunteer Japanese students (a man and woman) in a soundproof room (RION, AT-81) via a condenser microphone (Blue, Yeti USB microphone) and Praat Version 6.1.21 (16-bit, 44.1 kHz sampling rate, mono)^39^. Based on previous studies^40,41^, we selected four nonsense words (“moma,” “bamu,” “kipi,” and “kuhtay”) and also four nonsense words (“mamo,” “muba,” “piki,” and “taykuh”) that replaced the syllables before and after as speech stimuli, for a total of 16 speech stimuli (two speakers × eight nonsense words). “kipi,” “piki,” “kuhtay,” and “taykuh” were defined as angular speeches and “moma,” “mamo,” “bamu,” and “muba” were defined as rounded speeches. Each stimulus was presented via headphones (Bose, QuietComfort 3 Acoustic Noise Cancelling Headphones).

Similar to in Experiment 1, participants evaluated the response keys on five dimensions (tactile impression: “soft,” “hard,” “fluffy,” “jagged,” “ease of key press”). Table 2 lists the average values calculated for each dimension. The same trend was observed as in Experiment 1. We conducted a paired two-sided *t*-test (Bonferroni-corrected, where *p* < .05 prior to correction). A significant difference was observed between the response keys for the tactile impression (jaggedness: *t*(15) = 64.235, *p* < .001, *d* = 16.059; fluffiness: *t*(15) = 53.694, *p* < .001, *d* = 13.423; softness: *t*(15) = 33.497, *p* < .001, *d* = 8.374; hardness: *t*(15) = 29.767, *p* < .001, *d* = 7.442, all the comparisons were significant at *p* < .01). However, no significance was observed for the ease of pressing the key (*t*(15) = 0.795, *p* = .439, *d* = 0.199). This result showed that one key had fluffy and soft features, whereas the other had jagged and hard features. Additionally, there were no notable differences between the evaluations of response keys in Experiments 1 and 2 (detailed statistical results in the supplement materials).

**Table 2:** Answers to questionnaires about response keys.

	Jaggedness	Fluffiness	Hardness	Softness	Ease of pressing key
Jagged key	8.813 (0.101)	1.188 (0.136)	8.563 (0.157)	1.313 (0.176)	5.938 (0.602)
Fluffy key	1.125 (0.085)	8.938 (0.063)	1.438 (0.182)	8.625 (0.155)	6.625 (0.499)

Standard errors are indicated within parentheses.

### Procedures

The experimental design was the same as in Experiment 1, except for the replacement of visual stimuli with speech stimuli. Participants completed four blocks of 48 trials after eight practice trials. However, participants were familiarized with 16 speech stimuli in the practice trials in only the first block. Each block was assigned to either a compatible (jagged/fluffy key-pressing response to an angular/rounded speech stimulus) or an incompatible condition (jagged/fluffy key-pressing response to a rounded/angular speech stimulus). In the experimental trials, 16 speech stimuli were randomly presented three times. In the practice trials, except for the first block, eight speeches (eight nonsense words of either gender) were presented randomly. Combination of stimulus types and voice sex, conditions, and arrangement of response keys were counterbalanced. A short break was provided between each block, during which the experimenter swapped the positions of the response keys for each condition.

## Results

Definitions of RT and pre-treatment were the same as those used in Experiment 1. Of the trials, 4.10% and 1.60% were excluded as errors and outliers, respectively. We calculated the IES to combine speed and accuracy. Figure 1b illustrates the average IES for each combination of speech sounds and tactile features. Responses of the jagged keys to angular speech stimuli were faster (*M*_RT_ = 746.494 ms, *M*_PC_ = 95.833%) than to rounded speech stimuli (*M*_RT_ = 807.114 ms, *M*_PC_ = 96.094%). In contrast, the responses of fluffy keys to rounded-speech stimuli were faster and more accurate (*M*_RT_ = 773.745 ms, *M*_PC_ = 97.786%) than those to angular speech stimuli (*M*_RT_ = 823.150 ms, *M*_PC_ = 94.010%). We conducted a 2 (speech sounds: angular vs. rounded) × 2 (tactile features: jagged vs. fluffy) repeated-measures ANOVA. Consequently, the interaction between speech sounds and tactile features was significant (*F*(1,15) = 7.032, *p* = .018, η_p_^2^ = 0.319). The main effect of speech sounds (*F*(1,15) = 0.764, *p* = .396, η_p_^2^ = 0.049) and tactile-quality feature (*F*(1,15) = 3.131, *p* = .097, η_p_^2^ = 0.017) were not significant. Since the interaction was significant, we conducted simple effects tests. Keypresses to the jagged key for the angular speeches were faster than those to the fluffy key for the angular speeches (*F*(1,15) = 7.421, *p* = .016, η_p_^2^ = 0.331). Keypresses to the fluffy key for the rounded speeches were faster than those to the fluffy key for the angular speeches (*F*(1,15) = 4.844, *p* = .044, η_p_^2^ = 0.244). Furthermore, keypresses to the jagged key for the angular speeches were faster than those to the jagged key for the rounded speeches (*F*(1,15) = 5.800, *p* = .029, η_p_^2^ = 0.279). However, there was no difference in the IES between the fluffy and jagged keys for the rounded speeches (*F*(1,15) = 3.770, *p* = .071, η_p_^2^ = 0.201).

## Discussion

Results of Experiment 2 revealed that the IES for pressing the jagged key for angular speech was lower than that for rounded speech. Furthermore, the IES for pressing the fluffy key for rounded speech was lower than that for angular speech. These results suggested a correspondence between sound-symbolic speech and tactile-quality features. Together with the results of Experiment 1, the angular/rounded stimulus corresponded to tactile-quality features of jaggedness/fluffiness, regardless of the input modality. Furthermore, although the results of Experiment 1 did not reveal a facilitation effect with the jagged key, those of Experiment 2 revealed a facilitation effect with both jagged and fluffy keys. This difference was likely because while the jagged key had a rounded macro 3D shape, which led to a correspondence between the shapes of visual stimuli and response key in Experiment 1, this shape-related correspondence did not occur in Experiment 2. This difference hinted that the angular/rounded sound-symbolic speech and angular/rounded tactile shape did not correspond in a speeded classification task (it may be necessary to mediate tactile-quality features, such as hardness and roughness).

### General discussion

Research on crossmodal correspondences related to tactile features has employed evaluation tasks for tactile objects or explicit matching tasks between tactile objects and other sensory features. These tasks included the experimenter-expectancy effect and the influence of the linguistic labels of rating scales^18,19^ and thus, it was difficult to interpret the correspondence showing in such tasks as implicit or non-arbitrary correspondence. In contrast, speeded classification tasks could minimize the influence of these factors and suggest more implicit modes of correspondences than tasks used in the previous studies^16–18^. Therefore, this study aimed to assess whether crossmodal correspondences between angularity and jaggedness occurred via speeded classification tasks without explicit judgment of tactile-quality features. We conducted a speeded classification task where participants pressed a left or right key with a jagged or fluffy surface in accordance with the angular or rounded visual stimuli in Experiment 1 and angular or rounded sound-symbolic speech in Experiment 2. We assumed that the stimulus angularity/roundedness corresponded to the jaggedness/ fluffiness of the pressing key and the correct-positioned keypress was facilitated. In Experiments 1 and 2, we demonstrated that keypresses directed toward the jagged key were fast and accurate via a presentation of angular visual shapes or speech sounds, whereas keypresses directed toward the fluffy key were fast and accurate via a presentation of rounded visual shapes or speech sounds. These results suggested crossmodal correspondences between visual and speech angularity/roundedness and tactile jaggedness/fluffiness of response keys. Furthermore, the stimulus with angular/rounded features facilitated the pressing of the response key with the corresponding tactile-quality feature of jaggedness/fluffiness, regardless of the input modalities (visual and auditory). Moreover, the tactile-quality features of response keys were task-irrelevant because we instructed the participants to press the left or right spatially arranged response keys and focusing on the key’s tactile features was not necessary. Therefore, participants might learn jagged or fluffy impressions of key’s surface by pressing the left or right response keys through practice trial or during the task, and thus acquire the mapping between the spatial positions of keys and tactile-quality features. Specifically, the fast and accurate response for the correct-positioned key suggested that angular/rounded stimuli (visual shape and sound-symbolic speech) primed the expected sensory outcomes of jagged/fluffy impressions associated with the left or right response keys via crossmodal correspondences, which facilitated motor responses. We additionally analyzed whether participants performed better over time after learning the mapping and found that the facilitation effect was larger in later trials than in earlier trials in Experiment 1 (this result was not shown in Experiment 2; detailed information in supplementary material). Furthermore, previous studies used speeded classification tasks to reveal the correspondence between the tactile features of response keys and sensory features of input stimuli and focused on object properties, such as the size and weight of response keys. We addressed tactile-quality features, such as jaggedness. This study suggested that the physical properties of the response keys related to motor responses and their qualitative properties were incorporated into the ongoing process of stimulus-response and corresponded to the features of stimuli.

Examining the cognitive models that can explain these findings is important. Previous studies argued that correspondence emerged from interactions among the dimensions of connotative meaning^11,27,42^. This idea was suitable for explaining the correspondence between sensory features. However, this explanation did not include the processing of the sensory outcomes of response and facilitation of motor action. Therefore, we interpreted these results in combination with the Theory of Event Coding (TEC)^43,44^ and crossmodal correspondence as TEC could explain motor processing. The TEC claims that perception and response are identical processes controlled by the same codes (event files) composed of integrated processing of all stimuli, response, sensory outcomes of response, and response key information. It supports that the task-irrelevant jaggedness of the response key is incorporated into the common coding of other processes on the angularity of stimulus and position of the response key. In this experimental task, the left-/right-response event file included a stimulus feature (angular or rounded), key position (left or right), and tactile-quality feature code (jagged or fluffy), among others (although other feature codes might be included, they are omitted for this explanation). For example, in the compatible condition where participants were instructed to press the left response key with a jagged tactile-quality feature in response to an angular stimulus, the left-response event file included the angular stimulus feature, left key position, and jagged tactile-quality feature codes. The presentation of the angular stimulus activated the angular stimulus feature and left key position codes and additionally activated the jagged tactile-quality feature code through crossmodal correspondence. Since all these feature codes were included in the left-response event file, the execution of the left response proceeded smoothly based on crossmodal correspondence. Conversely, in the incompatible condition where participants were instructed to press the left response key with a fluffy tactile-quality feature in response to an angular stimulus, the left-response event file included the angular stimulus feature, left key position, and fluffy tactile-quality feature codes; however, it did not include the jagged tactile-quality feature code. Although the presentation of the angular stimulus activated the angular stimulus feature and left key position codes, the fluffy tactile-quality feature code contained in the left-response event file did not activate. Instead, the jagged tactile-quality feature code included in the right-response event file was activated through crossmodal correspondence. Consequently, conflict occurred between the left- and right-response event files, which led to a delay in the left key press response. Therefore, the angular/rounded stimulus activated the jagged/fluffy tactile feature code within the event file through crossmodal correspondence, which altered the event file’s weight, and facilitated or inhibited the left/right key press responses. This interpretation could explain our results and also previously-reported correspondences (visual brightness-tactile weight, size key:^17,27^; auditory pitch-visual spatially key:^45^; visual space-vocal pitch:^46^). Further investigations should identify the neural mechanisms supporting the reported correspondences. While there was distributed processing on single material property specific to a single modality^47,48^, several studies have claimed that the areas of the brain, which recognize material properties might be shared between modalities^49,50^. Some neuroimaging studies stated that the posterior superior temporal sulcus (pSTS), inferior frontal gyrus (Broca’s area), and middle occipital gyrus (MOG) are associated with sound symbolism^51–53^. In contrast, others claimed that the insula and medial superior frontal gyrus (SFG) contributed to sound symbolism between visually presented sound symbolic words and tactile softness^54^. Therefore, sound symbolism between vision and audition and vision and touch may stem from various multisensory areas of the brain. These areas partially overlap with the object’s surface perception structure and speech production networks (surface perception network:^55–58^; speech production network:^59–61^). Thus, multisensory interactions across these networks may share information on visual or auditory angularity and tactile jaggedness, and potentially result in correspondences. This speculation aligns with the idea that corresponding information is expressed at a more abstract level^62^. However, opinions differ on the abstract representation^63,64^, and convincing empirical evidence is still required.

We assume that the reported crossmodal correspondences were acquired through two conceivable mechanisms. First, internalization of statistical relationships. Several crossmodal correspondences can be explained by the correlation between pairs of sensory features in the natural environment^65^. Use of the statistical regularities of the natural environment to determine which possible sensory features correspond to each other may be plausible. For example, an object’s resonant frequency is related to its mass and stiffness^66,67^. People might associate high/low pitch with small/large size (since an object’s mass and size are strongly correlated if its density is constant) by internalizing this relationship. Regarding this study, hard materials have high elasticity/stiffness. Therefore, hard objects tend to have high resonant frequency and are able to maintain an angular shape, even when external forces are applied, compared with soft objects^68^. These statistical relationships might help explain the correspondences shown in this study. Additionally, several studies suggested that material properties, such as the hardness and roughness of objects, were represented in the same brain regions, regardless of the input modality, and evaluations related to these properties may be shared^49,50^. It also supported the correspondence between tactile features and other sensory features. Second, the mediation of speech production. Correspondences shown in Experiment 2 could be interpreted as a type of sound symbolism between speech sounds (fricative/sonorant syllables) and tactile-quality features (jaggedness/fluffiness). Sound symbolism is ordinarily explained as the mediation of feelings of turbulent airflow during the production of angular speech, including fricative consonants^69,70^. Several studies demonstrated that stimuli suggestive of speech elicited covert articulation, regardless of whether speech information was provided as auditory or visual stimuli^71,72^. When people pronounce angular-sounding words, such as “kipi” that contained fricative consonants, the turbulent airflow was caused by a constriction within the oral cavity. In contrast, when people pronounced rounded-sounding words, such as “moma” that contained sonorant consonants, no constriction occurred, and the airflow flowed smoothly. Turbulent airflow may induce a tactile analogous feeling of jaggedness, roughness, and hardness (relationship between surface and airflow:^73^). People may implicitly rely on such cues in articulation to associate themselves with other sensory features. Hence, people may form correspondences by associating angular shapes, angular-sounding words, and even jaggedness with feelings that occur in speech production within sensorimotor processing. However, a recent study explained sound symbolism regarding simple acoustic features rather than of articulatory features^74^. Tactile-related sound symbolism could also be explained by the correspondence between acoustic features (pitch and spectrum) and tactile features rather than articulatory features^75–77^. Future investigations should examine whether tactile-related sound symbolisms can arise even with unpronounceable sounds and distinguish the background mechanisms between an articulatory-based one and acoustic-based one.

In this study, the surface of the response keys was covered with pumice stones to express jaggedness and a piece of blanket to express fluffiness. From the questionnaire results, we confirmed that each response key effectively expressed the targeted tactile-quality features (jaggedness and fluffiness). However, the results indicated that the jagged and fluffy keys also expressed hardness and softness, respectively. Previous studies suggested that tactile information was conveyed from a single material/object^28^, and was expressed by three factors: hardness, roughness, and warmness^78^. We focused on jaggedness and hardness during stimulus manipulation. Roughness is perceived as multisensory^79^ and may also have influenced the present phenomenon, although we did not ask the participants regarding the roughness of the response keys. The tactile cue of roughness was also sufficient to provide an object’s geometrical shape information^80^. Thus, we could not exclude the possibility that factors, such as the roughness or shape of the tactile surface of response keys, which were not focused on, influenced the correspondences. Future studies should examine these results in greater detail by introducing appropriately controlled response keys with tactile features. Additionally, we determined the sample size of both the experiments (*n* = 16) based on the assumption of a large effect size from the results of our pilot study. However, we actually obtained a smaller effect size in the results of these experiments than that of the prediction. The present sample size was relatively small compared with previous studies with similar purposes and experimental paradigms. Previous studies had at least 20 participants^17,27,81^. This is another methodological limitation and should be considered in future research.

This study revealed that presenting angular/rounded stimuli facilitated keypresses on the side of the response keys that corresponded to the jagged/fluffy tactile-quality features. These results indicated correspondences between angularity and tactile-quality features (jaggedness and fluffiness) in the visual-tactile and auditory-tactile dimensions, regardless of task-irrelevance of tactile-quality features. Moreover, this study showed the correspondences via speeded classification tasks, and thus, we could minimize the influence of experimenter-expectancy effect and linguistic labels of rating scales. In addition, we suggest more implicit modes of correspondences than the tasks with explicit judgment of tactile features used in previous studies. Furthermore, our findings suggested that tactile-quality features of the response keys related to motor responses were incorporated into an ongoing process of stimulus-response and corresponded to the features of stimuli. These findings support the idea that the representation of tactile-quality features, visual shape, and speech sounds are shared at the abstract level^62^, which facilitates motor processing^44^. However, we could not exclude the possibility that other tactile features, such as the roughness and shape of response keys, influenced the correspondences. This should be considered in future research.

## Electronic supplementary material

Below is the link to the electronic supplementary material.

Supplementary Material 1

## Data Availability

The current experimental data are available from the corresponding author upon reasonable request.
