# Supplementary material for: Crossmodal correspondences between visual and speech angularity and tactile jaggedness of response key
Source: Sci Rep. 2024 Nov 13;14:27806. doi: 10.1038/s41598-024-79400-4 (PMC11561357; doi:10.1038/s41598-024-79400-4)
Supplement: Supplementary file 1 — Supplementary Material 1 [file 41598_2024_79400_MOESM1_ESM.docx]

# Supplementary Materials

## Pilot Study

We recruited 18 undergraduate students from Ritsumeikan University who reported normal or corrected-to-normal vision for the pilot study (*M*_age_ = 21.4, 11 males and seven females). The stimuli and experimental procedures were the same as those in Experiment 1. All participants in the pilot study did not participate in Experiments 1 and 2.

Similar to in Experiments 1 and 2, error trials and outlier trials were excluded, and the Inverse Efficiency Score (IES: [1]) was calculated for each combination of the visual and tactile-quality features to combine speed and accuracy (Supplementary Figure 1). IES was calculated by dividing the average reaction times (RT) by the proportion of correct responses (1-error rates). In tasks that required both speed and accuracy, experimental effects may appear in RT or error rate for some participants owing to the speed-accuracy trade-off. Ignoring one measure and drawing conclusions based solely on another measure can lead to misinterpretation. The IES addressed this issue by weighting speed according to accuracy. A lower IES indicated a quicker and more accurate response. We predicted that the IES would be lower for angular/rounded stimuli paired with jagged/fluffy keypresses than for the opposite combinations. A two-way analysis of variance (ANOVA) was conducted on the averaged IES, with visual shapes (2: angular or rounded) and tactile-quality features of response keys (2: jaggedness or fluffiness). Consequently, a significant interaction was revealed (*F*(1,17)=30.649, *p*<.001, η_p_^2^=.643). Both the main effects of visual shapes (*F* (1, 7)=1.600, *p*=.223, η_p_^2^=.086), and tactile-quality features (*F* (1,17)=0.640, *p*=.435, η_p_^2^=.036) were not significant. Since the interaction was significant, we conducted simple main effects tests, and all simple main effects were significant. Responses to fluffy keys were faster and more accurate than to jagged keys when presented with rounded stimuli (*F*(1,17)=25.442, *p*<.001, η_p_^2^=.600). Furthermore, responses to jagged keys were faster and more accurate than to fluffy keys when presented with angular stimuli (*F*(1,17)=24.292, *p*<.001, η_p_^2^=.588). Additionally, the responses to fluffy keys were faster and more accurate for rounded stimuli compared with angular stimuli (*F* (1,17)=12.176, *p*=.003, η_p_^2^=.417). Responses to jagged keys were faster and more accurate for angular stimuli compared with rounded stimuli (*F*(1,17)=24.841, *p*<.001, η_p_^2^=.594). The effect size of the interaction (*f*=0.514) observed in the pilot study exceeded Cohen's criterion for a large effect size (*f*=0.400). Thus, a large effect size was assumed for the sample size design in the main experiment.

Supplementary Figure 1


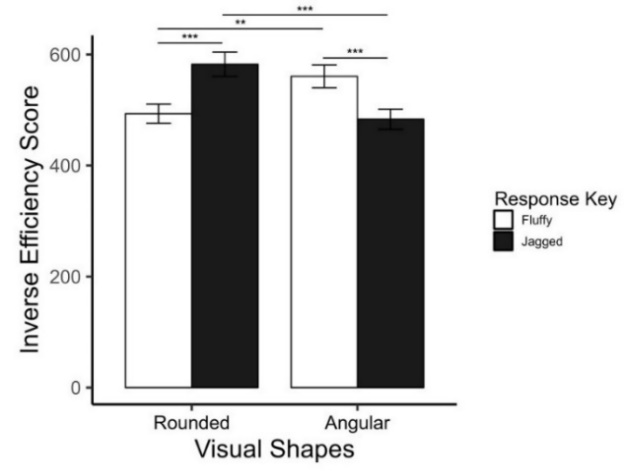


Mean IES for visual shapes × tactile-quality features of response keys. Error bars indicate standard errors. Asterisks indicate significant differences (**: *p*<.01; ***: *p*<.001).

##
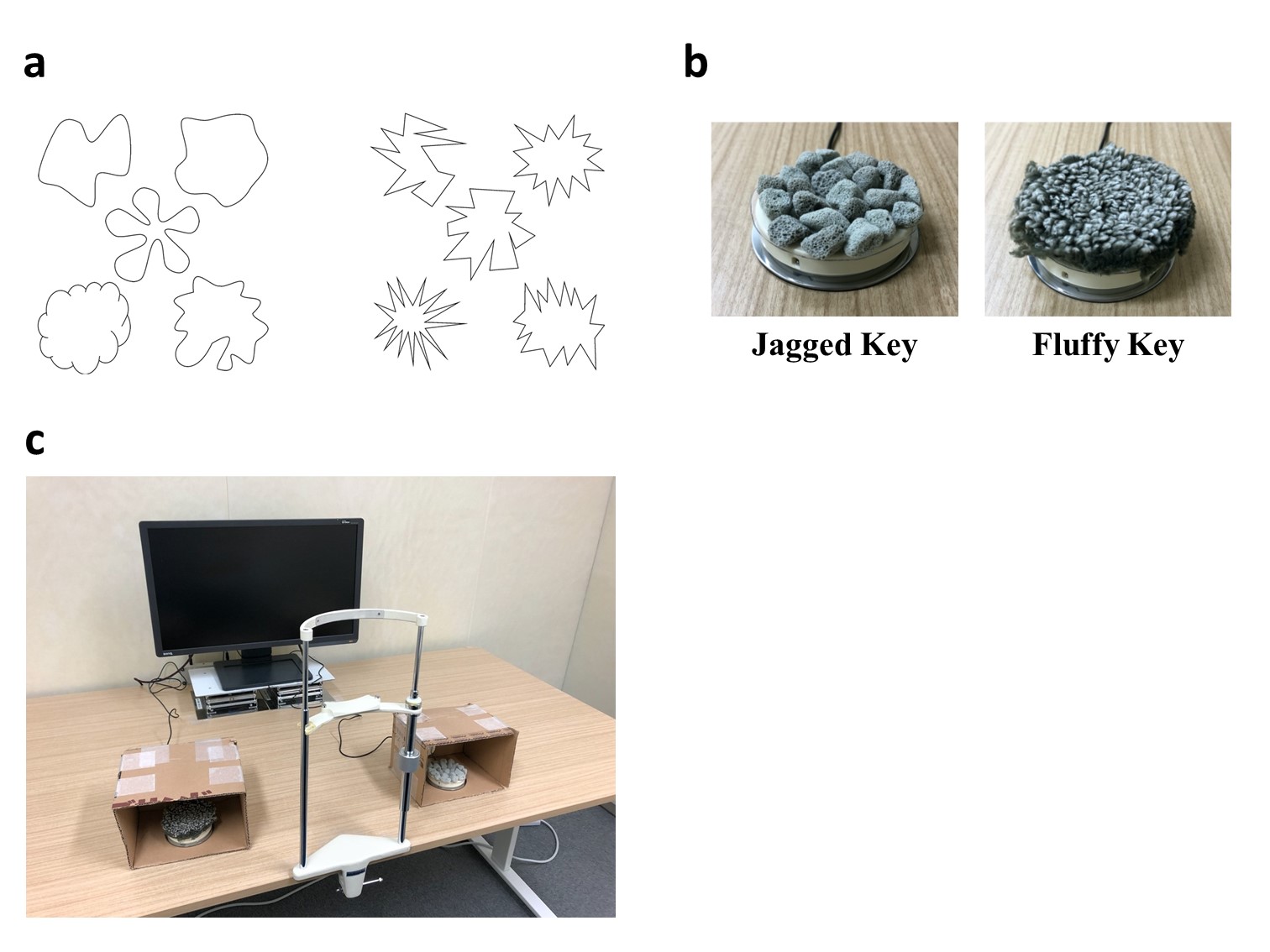
Visual stimuli, response keys, and experimental environment

Supplementary Figure 2

(a) Visual stimuli used in Experiment 1. (b) response keys with the tactile-quality features (jagged or fluffy). The key shown on the left and right is a jagged and fluffy key, respectively. The same response keys were used in both Experiments 1 and 2. (c) Experimental environment. In the actual experiments, the participants could not see the keys because a black cloth was put over the cardboard boxes.

## Learning effect in correspondence

### Improvement in performance over time

In Experiments 1 and 2, all the trials were divided into four blocks, and each block assigned either compatible or incompatible conditions. The first two blocks and subsequent two blocks were regarded as the first and second block, respectively. To examine the improvement in performance according to learning the association, we compared the IES differences between the compatible and incompatible conditions in the first and second block (a paired two-sided *t-*test). If crossmodal correspondences became stronger over time, the IES differences in the second block would be higher than that in the first block. In Experiment 1, the IES differences in the second block were higher than that in the first block (*t*(15)=2.398, *p*=.030, *d*=0.599). In Experiment 2, no IES differences were observed between the first and second block (*t*(15)=0.449, *p*=.660, *d*=0.112). These results suggested that participants learned the correspondence during the task and correspondence progressed over block, in particular for the correspondence between visual shape and tactile-quality features.

Supplementary Figure 3


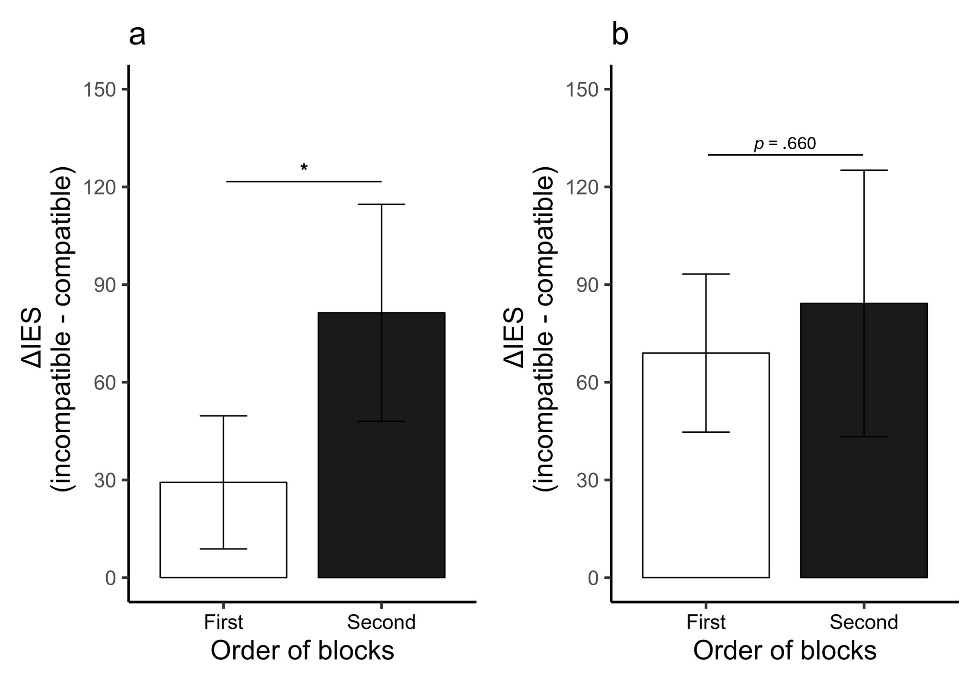


Mean IES differences between the first and second blocks in Experiments 1 (a) and 2 (b). Error bars indicate standard errors. Asterisks indicate significant differences (*: *p*<.05).

### Influence of the FIRST condition on the subsequent performance

Previous studies reported that an observed crossmodal correspondence was weak when participants experienced many incompatible trials. Conversely, it was strong when they experienced many compatible trials [2]. We examined the influence of previous condition on the strength of the crossmodal correspondence. In our experiments, half of participants first experienced a compatible condition (C-First sequence), and the others first experienced an incompatible condition (I-First sequence). We compared the differences in IES between the conditions (Welch’s *t*-test: order of conditions {C-First or I-First}, Supplementary Figure 4). Results revealed no significant effect of the first condition in both the experiments (Experiment 1: *t*(9.378)=0.599, *p*=.563; Experiment 2: *t*(13.873)=0.883, *p*=.392). These results suggested that the correspondences observed in this study would not be reflected by relatively small learning effect and were stable and robust. However, please note that our sample size was not designed to examine the effect of the preceding condition. Hence, this effect could occur with an increased sample size. Further investigations are required on the effects of preceding conditions.

Supplementary Figure 3


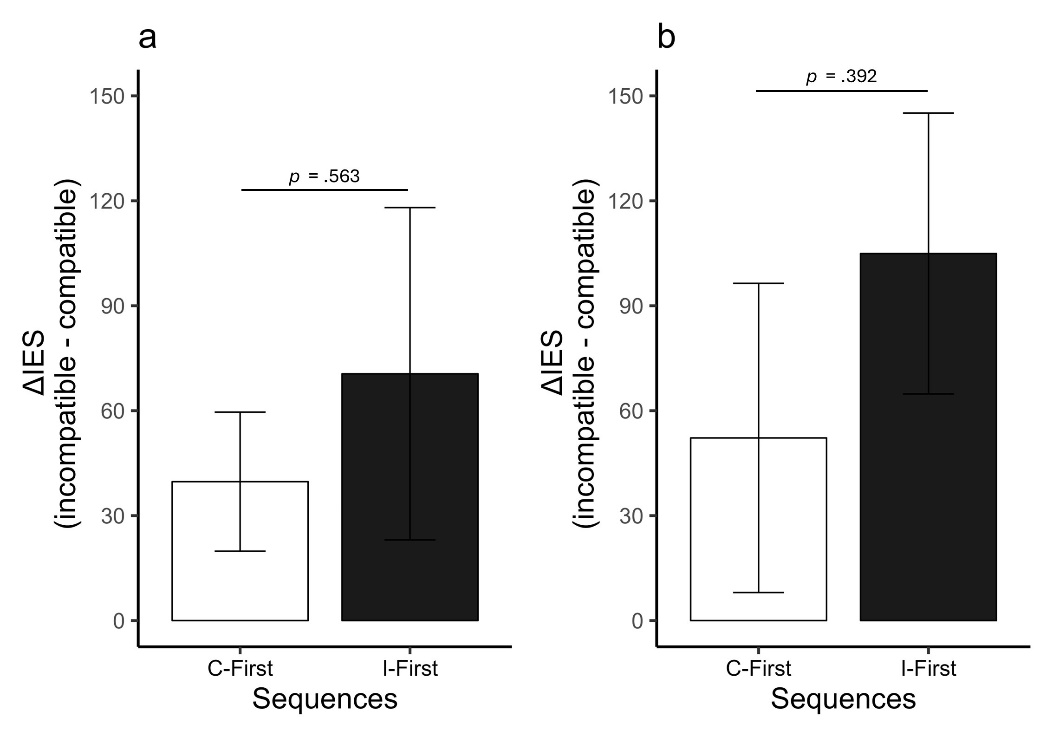


Mean IES differences between the experimental conditions for a sequence of conditions in Experiments 1 (a) and 2 (b). Error bars indicate standard errors.

## Comparing the evaluation of response keys between experiments

To confirm whether participants consistently evaluated the tactile-quality features (“soft,” “hard,” “fluffy,” and “jagged”) and ease of pressing the key in Experiments 1 and 2, we compared the evaluation scores between experiments for each item via Welch’s *t*-tests (Bonferroni-corrected, all comparisons were significant at *p*<.005). Results revealed no significant differences in any item (softness: *t*(15)=1.775, *p*=.096; hardness: *t*(20)=2.216, *p*=.039; fluffiness: *t*(15)=1.379, *p*=.188; jaggedness: *t*(29)=0.473, *p*=.640; ease of pressing key: *t*(30)=1.638, *p*=.112 in jagged key; softness: *t*(30)=0.910, *p*=.370; hardness: *t*(28)=1.101, *p*=.280; fluffiness: *t*(21)=0.835, *p*=.413; jaggedness: *t*(15)=1.464, *p*=.164; ease of pressing: *t*(30)=0.272, *p*=.788 in fluffy key). These analyses confirmed that the manipulations of the response keys were effective in both experiments.

# References

1. Townsend, J. T. & Ashby, F. G. *Stochastic modeling of elementary psychological processes* (Cambridge University Press, 1983).
2. Corballis, P. M. & Gratton, G. Independent control of processing strategies for different locations in the visual field. *Biol. Psychol.* **64**, 191-209 (2003).
